# Associations of pigmentary and naevus phenotype with melanoma risk in two populations with comparable ancestry but contrasting levels of ambient sun exposure

**DOI:** 10.1111/jdv.15680

**Published:** 2019-06-07

**Authors:** A.E. Cust, M. Drummond, D.T. Bishop, L. Azizi, H. Schmid, M.A. Jenkins, J.L. Hopper, B.K. Armstrong, J.F. Aitken, R.F. Kefford, G.G. Giles, F. Demenais, A.M. Goldstein, J.H. Barrett, P.A. Kanetsky, D.E. Elder, G.J. Mann, J.A. Newton‐Bishop

**Affiliations:** ^1^ Cancer Epidemiology and Prevention Research Sydney School of Public Health The University of Sydney Sydney Australia; ^2^ Melanoma Institute Australia The University of Sydney Sydney Australia; ^3^ Section of Epidemiology and Biostatistics Leeds Institute of Cancer and Pathology University of Leeds Leeds UK; ^4^ School of Mathematics and Statistics The University of Sydney Sydney Australia; ^5^ Centre for Cancer Research Westmead Institute for Medical Research The University of Sydney Sydney Australia; ^6^ Centre for Epidemiology and Biostatistics Melbourne School of Population and Global Health The University of Melbourne Melbourne Australia; ^7^ Viertel Centre for Research in Cancer Control Cancer Council Queensland Brisbane Australia; ^8^ Macquarie University Health Sciences Centre Macquarie University Sydney Australia; ^9^ Cancer Epidemiology Centre Cancer Council Victoria Melbourne Australia; ^10^ Genetic Variation and Human Diseases Unit UMR‐946 INSERM Université Paris Diderot Université Sorbonne Paris Cité Paris France; ^11^ Human Genetics Program Division of Cancer Epidemiology and Genetics National Cancer Institute Bethesda MD USA; ^12^ Cancer Epidemiology Program Moffitt Cancer Center Tampa FL USA; ^13^ Department of Pathology and Laboratory Medicine University of Pennsylvania Philadelphia PA USA

## Abstract

**Background:**

People at high risk of developing melanoma are usually identified by pigmentary and naevus phenotypes.

**Objective:**

We examined whether associations of these phenotypes with melanoma risk differed by ambient sun exposure or participant characteristics in two population‐based, case–control studies with comparable ancestry but different ambient sun exposure.

**Methods:**

Data were analysed from 616 cases and 496 controls from the Australian Melanoma Family Study and 2012 cases and 504 controls from the Leeds (UK) case–control study. Questionnaire, interview and dermatological skin examination data were collected using the same measurement protocols. Relative risks were estimated as odds ratios using unconditional logistic regression, adjusted for potential confounders.

**Results:**

Hair and skin colour were the strongest pigmentary phenotype risk factors. All associations of pigmentary phenotype with melanoma risk were similar across countries. The median number of clinically assessed naevi was approximately three times higher in Australia than Leeds, but the relative risks for melanoma associated with each additional common or dysplastic naevus were higher for Leeds than Australia, especially for naevi on the upper and lower limbs. Higher naevus counts on the head and neck were associated with a stronger relative risk for melanoma for women than men. The two countries had similar relative risks for melanoma based on self‐reported naevus density categories, but personal perceptions of naevus number differed by country. There was no consistent evidence of interactions between phenotypes on risk.

**Conclusions:**

Classifying people at high risk of melanoma based on their number of naevi should ideally take into account their country of residence, type of counts (clinical or self‐reported), body site on which the naevus counts are measured and sex. The presence of naevi may be a stronger indicator of a genetic predisposition in the UK than in Australia based on less opportunity for sun exposure to influence naevus development.

## Introduction

Melanoma rates have been increasing,^1^ despite being a largely preventable disease.^2^ People at high risk of developing melanoma are often identified by pigmentary and naevus phenotypes. Melanocytic naevi predominantly originate in childhood, and their development is influenced by sun exposure and genetic factors.^3^, ^4^, ^5^, ^6^, ^7^ A person's number of naevi may change over time with age and sun exposure,^5^, ^8^, ^9^, ^10^, ^11^ which could contribute to different magnitudes of association between naevi and melanoma risk by region, age or sex.

Although pigmentary and naevus phenotypes are established risk factors for melanoma, the magnitude of these associations may differ by geographical region, participant characteristics and study methodology.^12^, ^13^, ^14^, ^15^ One previous clinic‐based study with 300 cases and 325 controls suggested that atypical naevi were a stronger risk factor for melanoma in the United Kingdom (UK) than in Australia^9^; however, few population‐based data are available. We also have limited understanding of possible interactions between different risk factors.^12^, ^13^

To address these knowledge gaps and overcome limitations of previous studies, we examined the association of pigmentary and naevus phenotype with melanoma risk in two large, population‐based case–control studies under the auspices of the melanoma genetics consortium (GenoMEL, http://www.genomel.org). The studies were conducted using the same measurement protocols implemented as far as feasible in an identical manner and conducted in populations with similar ethnic backgrounds (Australia and the UK) but very different ambient sun exposure.

## Materials and methods

### Study samples

The Australian Melanoma Family Study was a multi‐centre, population‐based, case–control family study of invasive cutaneous melanoma diagnosed between ages 18–39 years. The study design, recruitment, data collection and participant characteristics have been described.^16^ Recruitment of case (*n* = 629) and control (*n* = 535) participants was locally coordinated in Sydney, Melbourne, and Brisbane, Australia. Cases were identified from population‐based state cancer registries, diagnosed between 1st July 2000 and 31st December 2002 at ages 18–39 years with incident, histopathologically confirmed, first primary invasive cutaneous melanoma. Participation was 76% of those contactable. Population controls were aged between 18–39 years at the time of approach and had no history of invasive or in situ melanoma. They were selected from the electoral roll (registration to vote is compulsory for Australians aged ≥18 years) and were frequency‐matched to cases by city, age and sex. Participation was 42% of those contactable. In addition, spouse/partner or friend controls were recruited through nomination by a case. They had to be at least 18 years of age and have no history of invasive or in situ melanoma; there were no other age, sex or residency restrictions. A spouse or friend was nominated as a potential control subject by 59% of cases, and participation was 80% of those nominated.

The Leeds case–control study recruited population‐based incident histopathologically confirmed invasive melanoma cases (*n* = 2184), aged between 18–82 years, and living in a geographically defined area of Yorkshire and the Northern region of the UK.^17^, ^18^ The cases were identified through clinicians, pathology registers and the cancer registry to ensure maximal ascertainment (67% participation). Between September 2000 and June 2003, all people with invasive melanoma were invited to participate. From July 2003 to September 2011, only cases with Breslow thickness ≥0.75 mm were invited, in order to enrich the cohort to observe clinical outcomes. Population‐ascertained controls were identified by the cases’ family doctors as not having cancer and were randomly invited from individuals who were matched by sex and age (55% participation, 513 recruited).

Approval for the study was obtained from the ethics committees of the coordinating centres in Australia and Leeds and the cancer registries. All participants provided written‐informed consent.

### Self‐reported pigmentary phenotype and naevus counts

Participants completed a questionnaire in which they reported skin colour, eye colour, natural hair colour at age 18, freckling as a child and adult,^19^ ability to tan, propensity to sunburn, usual tanning and sunburn response to prolonged or repeated exposure of skin to sunlight in summer, number of naevi covering their body (described pictorially as none, few, some, many) and were asked to have someone count the number of all moles on their back (using picture guides).^16^

We created a pigmentation score using factor analysis: this contained skin colour, eye colour, childhood freckling and skin phototype (see Data S1).

### Clinical assessment of pigmentary and naevus phenotype for a subset of participants

Clinical assessment was conducted by research nurses in the UK and by dermatology trainees in Australia. Assessors were jointly trained on the study protocol including recognizing and counting naevi according to international guidelines,^20^ and annual refresher courses were conducted jointly. Melanocytic naevi were defined as brown‐to‐black pigmented macules or papules which were reasonably well defined and darker in colour than the surrounding skin. Dysplastic (atypical) naevi were defined as having a macular component in at least one area in addition to at least three of the following: (i) ill‐defined border, (ii) size ≥5 mm, (iii) variegated colour, (iv) uneven outline and (v) erythema.^20^ Participants removed their clothing except for underpants and bra. Separate counts were made for melanocytic naevi of 2‐<5 mm and ≥5 mm, and clinically atypical naevi on different body sites but excluding scalp, breasts, buttocks and genitals. Naevi <2 mm were not counted to minimize confusion with freckles and lentigines.

Clinical assessment of naevi was completed for the first 1022 cases, and all population controls in Leeds, and by 73% of cases, 55% of population controls, and 67% of spouse or friend controls in Australia. Natural hair colour at age 18 and eye colour were also recorded using wig hair swatches and eye photographs for comparison.

### Self‐reported personal sun exposure

Comprehensive data on sun exposure throughout life were collected by telephone interview, with the aid of a residence calendar. Questions referred to the frequency of sunburn and time spent outdoors between 9 am and 5 pm separately for weekdays, weekends and holidays in warmer months and in cooler months.^18^, ^21^

### Statistical analysis

We excluded: relatives, participants who did not complete a questionnaire, Australian controls who were ≥45 years at interview (since all Australian cases were diagnosed <40 years), and participants missing either three or more key pigmentary phenotype variables, self‐report naevus density, summer holiday sun exposure or painful sunburn variables. The analysis dataset included 1112 participants (616 cases, 496 controls) from Australia and 2516 participants (2012 cases, 504 controls) from Leeds. Analyses of the associations between naevi and melanoma risk excluded participants with missing pigmentation score or hair colour, and analyses of the associations between clinically assessed naevus phenotype and melanoma risk were restricted to participants who had a clinical skin examination. Australian population controls (*n* = 237) and spouse/friend controls (*n* = 259) were combined into one control group for analysis, as done previously.^16^

Relative risks (RR) for melanoma were estimated as odds ratios (OR) and 95% confidence intervals (CI), using unconditional logistic regression. Minimally adjusted models included age (continuous), sex and city of recruitment (in Australia). Analyses of pigmentary phenotype were further adjusted for self‐reported naevus density, summer holiday sun exposure hours and painful sunburns; and analyses of naevus phenotype were further adjusted for pigmentation score and hair colour. We estimated the OR per standard deviation adjusted for age (5‐yr groups) and sex (OPERA method^22^) as a way of comparing the predictive strength of naevus number across Leeds and Australia while accounting for the countries’ different naevus, age and sex distributions.

To test whether the pigmentary and naevus phenotype associations with melanoma differed between Leeds and Australia, or by other factors, we added to the models a product term between the phenotype variable and country (or other factor), fitted as a one degree‐of‐freedom ordinal variable to test for interaction in the trend effect. Data were analysed using SAS version 9.4 (SAS Institute, Cary NC), and statistical significance was inferred at two‐sided *P* <0.05. We reported the study according to STROBE guidelines for observational studies.

## Results

### Characteristics

Demographic characteristics are shown in Table 1. Both studies had a majority of female participants. They had a similar proportion of participants with self‐reported European ethnicity, but the Australian study had a higher proportion of Eastern European ethnic background. Excluding Eastern Europeans from our analyses did not materially impact results.

**Table 1:** Characteristics of cases and controls in the Australian Melanoma Family Study and Leeds case–control study

Characteristic	Australia *N* (%)	Leeds *N* (%)
**Total, cases and controls**	1112	2516
Cases	616 (55)	2,012 (80)
Controls	496 (45)	504 (20)
**Sex**		
Female	663 (60)	1,438 (57)
Male	449 (40)	1,078 (43)
**Age at diagnosis/interview (years)** †		
18–29	291 (26)	105 (4)
30–39	733 (66)	287 (11)
40–49	88 (8)	445 (18)
50–69	0 (0)	1,335 (53)
≥70	0 (0)	344 (14)
**Ethnic background** ‡		
English	676 (61)	2,340 (93)
Scottish, Irish, Welsh	54 (5)	120 (5)
Other Northern European	49 (4)	14 (1)
Southern European	12 (1)	6 (0)
Eastern European	251 (23)	4 (0)
Mixed/Other European	20 (2)	28 (1)
Non‐European	49 (4)	0 (0)
Missing	1	4

†Leeds cases and controls were unselected for age at diagnosis. In Australia, all cases were <40 years at diagnosis and all population controls were <40 years when ascertained; cases and controls could be up to age 44 years at interview for this analysis.

‡Self‐reported.

### Pigmentary phenotype and melanoma risk

The associations of self‐reported and clinically assessed pigmentary phenotype factors with melanoma risk were similar across countries (Table 2). Pigmentation score was associated with an approximately twofold increased melanoma risk for the highest vs. lowest tertile. Both studies observed a threefold to fourfold increased risk of melanoma for those with red hair, and a twofold increased risk for those with fair or blonde hair, compared to those with dark brown or black hair. Very fair skin more than doubled risk compared with having olive or brown skin. The results remained consistent in the analyses adjusted for other risk factors and excluding naevus count density from the multivariable models had minimal impact on the risk estimates.

**Table 2:** Association of pigmentary phenotype with melanoma risk in the Australian Melanoma Family Study and Leeds case–control study

Pigmentary phenotype†	Australia (*N* = 1112)				Leeds (*N* = 2516)				*P*‐int¶
	Case *N* (%)	Control *N* (%)	OR (95% CI)‡	Adjusted OR (95% CI)§	Case *N* (%)	Control *N* (%)	OR (95% CI)‡	Adjusted OR (95% CI)§	
**Hair colour**									
Dark brown/black	160 (26)	200 (40)	1.00	1.00	822 (41)	288 (57)	1.00	1.00	0.37
Light brown	244 (40)	208 (42)	1.41 (1.05, 1.88)	1.26 (0.93, 1.70)	537 (27)	119 (24)	1.62 (1.27, 2.06)	1.61 (1.25, 2.06)	
Fair or Blonde	142 (23)	64 (13)	2.64 (1.82, 3.84)	2.38 (1.61, 3.51)	398 (20)	68 (13)	2.05 (1.54, 2.75)	1.99 (1.48, 2.69)	
Red	70 (11)	24 (5)	3.41 (2.03, 5.75)	4.21 (2.44, 7.27)	253 (13)	29 (6)	3.10 (2.06, 4.66)	3.01 (1.99, 4.57)	
**Eye colour**									
Brown or Black	111 (18)	119 (24)	1.00	1.00	322 (16)	94 (19)	1.00	1.00	0.62
Green or Hazel	224 (36)	167 (34)	1.52 (1.08, 2.13)	1.35 (0.95, 1.93)	605 (30)	182 (36)	0.98 (0.73, 1.30)	0.97 (0.72, 1.30)	
Blue or Grey	279 (45)	206 (42)	1.57 (1.13, 2.17)	1.49 (1.06, 2.09)	1075 (54)	228 (45)	1.40 (1.07, 1.84)	1.40 (1.06, 1.85)	
**Skin colour**									
Olive or Brown	62 (10)	92 (19)	1.00	1.00	149 (7)	60 (12)	1.00	1.00	0.30
Fair	430 (70)	336 (68)	1.92 (1.33, 2.76)	1.75 (1.20, 2.55)	1342 (67)	365 (72)	1.51 (1.09, 2.08)	1.48 (1.06, 2.08)	
Very fair	119 (19)	67 (14)	2.33 (1.48, 3.66)	2.29 (1.42, 3.68)	519 (26)	79 (16)	2.63 (1.79, 3.86)	2.54 (1.71, 3.79)	
**Freckles in childhood**									
None	116 (19)	127 (26)	1.00	1.00	543 (28)	187 (37)	1.00	1.00	0.28
Very few	185 (30)	163 (33)	1.17 (0.83, 1.64)	1.17 (0.82, 1.66)	571 (29)	131 (26)	1.46 (1.13, 1.89)	1.33 (1.02, 1.73)	
Few/Some	244 (40)	156 (32)	1.56 (1.12, 2.18)	1.69 (1.19, 2.39)	698 (35)	159 (32)	1.48 (1.15, 1.89)	1.35 (1.05, 1.75)	
Many	69 (11)	48 (10)	1.41 (0.89, 2.24)	1.55 (0.95, 2.52)	161 (8)	26 (5)	2.11 (1.34, 3.31)	2.06 (1.30, 3.29)	
**Freckles in adulthood**									
None	160 (26)	156 (32)	1.00	1.00	654 (33)	209 (42)	1.00	1.00	0.38
Very few	238 (39)	200 (40)	1.07 (0.79, 1.45)	1.03 (0.75, 1.40)	630 (31)	132 (26)	1.51 (1.17, 1.93)	1.38 (1.07, 1.78)	
Few/Some	175 (28)	119 (24)	1.25 (0.89, 1.75)	1.24 (0.87, 1.77)	609 (30)	144 (29)	1.35 (1.05, 1.73)	1.22 (0.94, 1.58)	
Many	42 (7)	20 (4)	1.62 (0.89, 2.95)	1.67 (0.89, 3.13)	108 (5)	18 (4)	1.87 (1.10, 3.18)	1.83 (1.06, 3.16)	
**General skin reaction to sun (skin phototype)**									
Sometimes/Never Burns	290 (47)	292 (59)	1.00	1.00	1276 (64)	375 (75)	1.00	1.00	0.88
Usually/Always Burns	322 (53)	200 (41)	1.55 (1.21, 1.99)	1.60 (1.23, 2.09)	726 (36)	127 (25)	1.69 (1.36, 2.11)	1.62 (1.28, 2.04)	
**Ability to tan from repeated exposure**									
Moderate/Deep Tan	359 (59)	331 (67)	1.00	1.00	542 (53)	336 (67)	1.00	1.00	0.13
Mild/No Tan	254 (41)	162 (33)	1.32 (1.02, 1.70)	1.33 (1.01, 1.74)	484 (47)	163 (33)	1.85 (1.48, 2.33)	1.88 (1.48, 2.39)	
**Propensity to sunburn**									
Mild/No burn	222 (36)	242 (49)	1.00	1.00	606 (59)	343 (69)	1.00	1.00	0.44
Severe/Painful burn	392 (64)	252 (51)	1.68 (1.31, 2.16)	1.69 (1.30, 2.20)	418 (41)	157 (31)	1.51 (1.20, 1.89)	1.47 (1.16, 1.86)	
**Clinically assessed hair colour**									
Black/Brown	176 (39)	172 (57)	1.00	1.00	424 (68)	217 (84)	1.00	1.00	0.55
Fair/Blonde	195 (43)	109 (36)	1.59 (1.15, 2.20)	1.48 (1.05, 2.08)	102 (16)	25 (10)	2.09 (1.31, 3.34)	2.14 (1.31, 3.49)	
Red	82 (18)	21 (7)	3.27 (1.90, 5.60)	3.58 (2.05, 6.27)	93 (15)	16 (6)	2.90 (1.66, 5.07)	3.19 (1.79, 5.66)	
**Clinically assessed eye colour**									
Brown	87 (19)	68 (23)	1.00	1.00	101 (11)	77 (15)	1.00	1.00	0.44
Green/Hazel	132 (29)	94 (31)	1.11 (0.72, 1.71)	1.06 (0.68, 1.65)	332 (35)	195 (39)	1.35 (0.95, 1.91)	1.31 (0.91, 1.88)	
Blue/Grey	231 (51)	140 (46)	1.44 (0.97, 2.14)	1.43 (0.95, 2.16)	515 (54)	229 (46)	1.85 (1.31, 2.59)	1.83 (1.29, 2.60)	
**Pigmentation score** ††									
Tertile 1	120 (20)	153 (31)	1.00	1.00	483 (25)	190 (38)	1.00	1.00	0.32
Tertile 2	188 (31)	145 (30)	1.55 (1.10, 2.18)	1.57 (1.11, 2.22)	629 (32)	170 (34)	1.40 (1.10, 1.80)	1.36 (1.06, 1.75)	
Tertile 3	296 (49)	188 (39)	1.83 (1.32, 2.52)	1.92 (1.38, 2.67)	839 (43)	141 (28)	2.40 (1.86, 3.10)	2.17 (1.67, 2.82)	

CI, confidence interval; OR, odds ratio.

Missing data for each variable (*N* for Australia, *N* for Leeds): hair colour (0, 2), eye colour (6, 10), skin colour (6, 2), freckling child (4, 40), freckling adult (2, 12), skin reaction to sun (8, 12), skin often exposed (6, 991), skin exposed (4, 992), clinically assessed hair colour (357, 1639), clinically assessed eye colour (360, 1067) and pigmentation score (22, 64).

†Phenotype variables were based on self‐report unless specified as clinically assessed.

‡Minimally adjusted models adjusted for age (continuous), sex and city of recruitment in Australia.

§Further adjusted for self‐reported naevus density, summer holiday sun exposure hours per day and painful sunburns to 40 years of age.

¶*P*‐value for the interaction between pigmentary phenotype variable and country (Australia/Leeds) using minimally adjusted models.

††Based on factor analysis (see Supplementary online material). Tertile cut‐points were based on the combined Australia/Leeds control distributions.

### Naevus phenotype and melanoma risk

**Figure 1:**
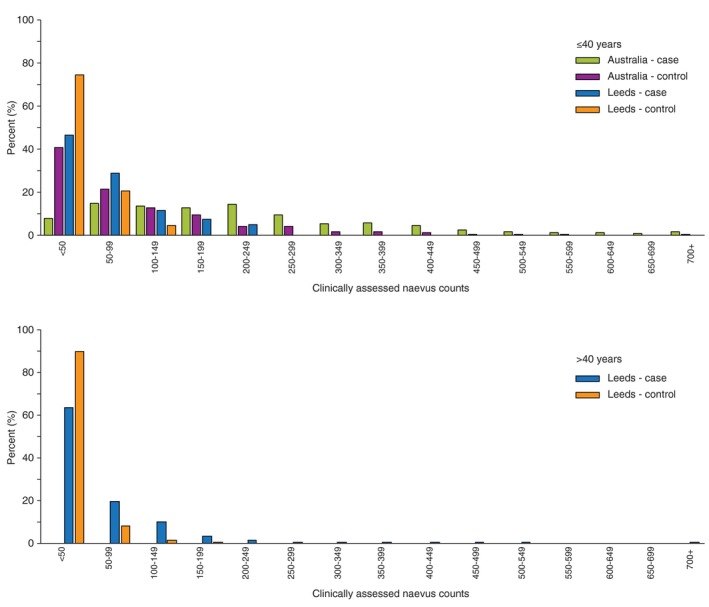
The distribution of clinically measured whole‐body naevus counts ≥2 mm for Australian cases, Australian controls, Leeds cases, Leeds controls, stratified by age ≤40, >40 years. The *x*‐axis represents the number of clinically assessed naevi, and the *y*‐axis represents the proportion of participants.

**Figure 2:**
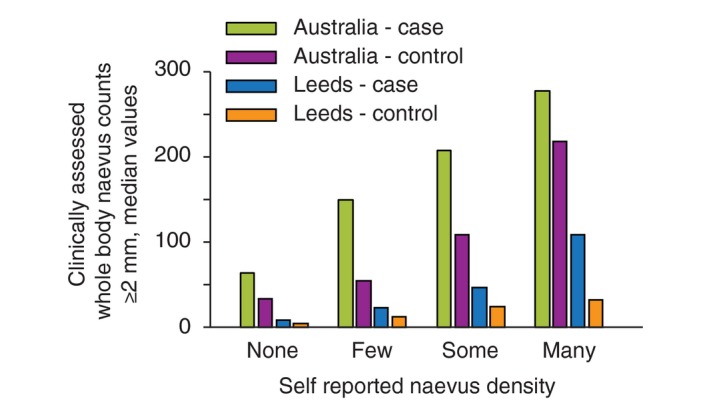
Comparison of self‐reported and clinically measured naevus counts (≥2 mm) in the Australian Melanoma Family Study and Leeds case–control study. The bar graph plots the median clinically measured naevus counts (*y*‐axis) according to self‐reported naevus density category (none, few, some, many) (*x*‐axis), separately for cases and controls in Australia and Leeds.

Cases had a significantly higher median number of naevi than controls, and Australian participants had more naevi than Leeds participants, even after accounting for the different age distributions (Fig. 1). These differences were apparent for both self‐reported naevi (Table 3) and clinically assessed naevi (Table 4). Participants’ perceptions of their own naevus density, when compared to clinical counts, differed by country and disease status (Fig. 2). For example, Leeds control participants who self‐reported ‘many’ naevi had a median of 34 naevi ≥2 mm diameter (interquartile range (IQR) 24–56), which was similar to Australian control participants who self‐reported ‘none’ (median 35, IQR 11–58). Nevertheless, the relative risks for melanoma associated with a higher self‐reported naevus density category were similar for Australia and Leeds (Table 3). Compared with those who self‐reported no naevi, those with ‘some’ naevi had an approximately threefold higher risk, and those with ‘many’ naevi had an approximately fivefold increased risk.

**Table 3:** Association of self‐reported naevus phenotype with melanoma risk in the Australian Melanoma Family Study and Leeds case–control study

Naevi	Australia (*N* = 1093)				Leeds (*N* = 2479)				*P*‐int§
	Case *N* (%) or median (IQR)	Control *N* (%) or median (IQR)	OR (95% CI)†	Adjusted OR (95% CI)‡	Case *N* (%) or median (IQR)	Control *N* (%) or median (IQR)	OR (95% CI)†	Adjusted OR (95% CI)‡	
**Self‐reported naevi**									
None	21 (3)	40 (8)	1.00	1.00	176 (9)	97 (19)	1.00	1.00	0.44
Few	172 (28)	237 (49)	1.30 (0.73, 2.32)	1.42 (0.78, 2.59)	771 (39)	252 (50)	1.67 (1.26, 2.23)	1.75 (1.30, 2.35)	
Some	264 (44)	158 (33)	3.05 (1.71, 5.45)	3.44 (1.88, 6.29)	718 (37)	118 (24)	3.29 (2.37, 4.56)	3.54 (2.53, 4.96)	
Many	147 (24)	51 (10)	5.17 (2.75, 9.72)	5.71 (2.96, 11.02)	296 (15)	34 (7)	4.67 (3.00, 7.29)	4.82 (3.07, 7.58)	
**Self‐reported naevi on the back** ¶									
Quartiles									
Quartile 1 (AMFS: 0–3; Leeds: 0–0)	96 (17)	136 (29)	1.00	1.00	300 (16)	123 (26)	1.00	1.00	0.82
Quartile 2 (AMFS: 4–8; Leeds: 1–3)	113 (19)	107 (23)	1.46 (0.99, 2.14)	1.52 (1.03, 2.26)	356 (19)	118 (25)	1.22 (0.91, 1.64)	1.23 (0.91, 1.67)	
Quartile 3 (AMFS: 9–19; Leeds: 4–10)	150 (26)	112 (24)	1.75 (1.21, 2.53)	1.85 (1.26, 2.71)	536 (29)	125 (27)	1.70 (1.27, 2.27)	1.81 (1.34, 2.43)	
Quartile 4 (AMFS: >=20; Leeds: >=11)	221 (38)	114 (24)	2.63 (1.85, 3.76)	2.79 (1.93, 4.03)	677 (36)	104 (22)	2.49 (1.83, 3.39)	2.71 (1.98, 3.71)	
**Continuous variables**									
Median (IQR) & OR per 1 naevi††	13 (6, 29)	8 (3, 19)	1.02 (1.01, 1.03)	1.02 (1.01, 1.03)	6 (2, 17)	3 (0, 10)	1.02 (1.01, 1.03)	1.02 (1.01, 1.03)	0.41
OR per adjusted SD increase in naevi‡‡			1.47 (1.27, 1.71)	1.50 (1.29, 1.74)			1.38 (1.19, 1.60)	1.42 (1.22, 1.65)	0.37

OR, odds ratio; CI, confidence interval; IQR, interquartile range; SD, standard deviation.

Missing data for each variable (*N* for Australia, *N* for Leeds): naevus density (3, 17), naevi on back (44, 140).

†Minimally adjusted models adjusted for age (continuous), sex, and city of recruitment in Australia.

‡Further adjusted for pigmentation score and hair colour.

§*P*‐value for the interaction between naevus phenotype and country (Australia/Leeds) using minimally adjusted models.

¶Quartile cut‐points were based on the country‐specific control distributions.

††OR per 1‐unit increase in naevus count modelled as a continuous variable.

‡‡OR per adjusted standard deviation, stratified by country (Australia/Leeds) and adjusted for age (5‐year groups) and sex, using the OPERA method.^22^

**Table 4:** Association of clinically assessed naevus phenotype with melanoma risk in the Australian Melanoma Family Study and Leeds case–control study

Naevi	Australia (*N* = 740)				Leeds (*N *= 1450)				*P*‐int§
	Case *N* (%) or median (IQR)	Control *N* (%) or median (IQR)	OR (95% CI)†	Adjusted OR (95% CI)‡	Case *N* (%) or median (IQR)	Control *N* (%) or median (IQR)	OR (95% CI)†	Adjusted OR (95% CI)‡	
**Naevi on the whole body ≥ 2 mm**									
***Categories***									
0–15	6 (1)	36 (12)	1.00	1.00	193 (20)	258 (52)	1.00	1.00	0.97¶
16–40	20 (5)	56 (19)	2.08 (0.74, 5.85)	1.44 (0.50, 4.19)	291 (31)	163 (33)	2.39 (1.82, 3.14)	2.48 (1.88, 3.29)	
41–60	33 (7)	42 (14)	5.02 (1.83, 13.76)	4.97 (1.79, 13.80)	148 (16)	40 (8)	4.91 (3.27, 7.37)	5.29 (3.49, 8.02)	
61–80	22 (5)	28 (9)	5.11 (1.75, 14.96)	4.75 (1.60, 14.13)	81 (9)	21 (4)	5.14 (3.05, 8.66)	5.40 (3.17, 9.20)	
81–100	21 (5)	17 (6)	9.06 (2.93, 27.98)	6.83 (2.15, 21.71)	66 (7)	5 (1)	17.47 (6.86, 44.47)	16.44 (6.42, 42.08)	
101–200	119 (27)	70 (24)	12.65 (4.86, 32.90)	10.91 (4.15, 28.70)	136 (14)	12 (2)	15.05 (8.03, 28.20)	14.84 (7.87, 28.01)	
≥201	222 (50)	48 (16)	35.98 (13.65, 94.82)	31.36 (11.75, 83.67)	36 (4)	0 (0)	n/a	n/a	
***Quartiles***									
Q1 (AMFS: 0–29; Leeds: 0–7)	19 (4)	73 (25)	1.00	1.00	75 (8)	134 (27)	1.00	1.00	0.12
Q2 (AMFS: 30–69; Leeds: 8–15)	56 (13)	76 (26)	3.29 (1.73, 6.25)	4.15 (2.10, 8.18)	118 (12)	124 (25)	1.68 (1.15, 2.46)	1.90 (1.28, 2.82)	
Q3 (AMFS: 70–155; Leeds: 16–29)	94 (21)	73 (25)	6.45 (3.42, 12.18)	7.36 (3.76, 14.42)	185 (19)	121 (24)	2.72 (1.88, 3.93)	3.03 (2.07, 4.43)	
Q4 (AMFS: >155; Leeds: >29)	274 (62)	75 (25)	20.10 (10.68, 37.83)	22.79 (11.65, 44.57)	573 (60)	120 (24)	8.37 (5.85, 11.98)	9.33 (6.43, 13.54)	
***Continuous variables***									
**Median (IQR) & OR per 1 naevi** ††									
Whole‐body naevi	201 (106, 308)	68 (30, 157)	1.01 (1.01, 1.01)	1.01 (1.01, 1.01)	40 (19, 81)	15 (7, 29)	1.03 (1.02, 1.03)	1.03 (1.02, 1.03)	<.0001
Head and neck naevi	14 (6, 24)	6 (2, 13)	1.06 (1.04, 1.08)	1.07 (1.05, 1.09)	3 (1, 6)	1 (1, 3)	1.15 (1.11, 1.20)	1.16 (1.11, 1.20)	0.0010
Trunk naevi	45 (23, 74)	18 (7, 43)	1.02 (1.02, 1.03)	1.02 (1.02, 1.03)	10 (4, 24)	5 (2, 11)	1.04 (1.03, 1.05)	1.04 (1.03, 1.06)	0.0003
Upper limbs naevi	76 (36, 121)	27 (12, 57)	1.02 (1.01, 1.02)	1.02 (1.01, 1.02)	11 (4, 22)	3 (1, 8)	1.09 (1.07, 1.11)	1.09 (1.07, 1.11)	<.0001
Lower limbs naevi	53 (26, 91)	16 (5, 41)	1.02 (1.01, 1.02)	1.02 (1.01, 1.02)	10 (4, 25)	3 (1, 7)	1.07 (1.05, 1.08)	1.06 (1.05, 1.08)	<.0001
**OR per adjusted SD increase in naevi** ‡‡									
Whole‐body naevi			2.62 (2.08, 3.30)	2.60 (2.06, 3.29)			3.09 (2.50, 3.81)	3.07 (2.48, 3.81)	0.20
Head and neck naevi			1.84 (1.50, 2.25)	1.99 (1.60, 2.47)			1.60 (1.39, 1.84)	1.62 (1.41, 1.87)	0.16
Trunk naevi			2.26 (1.82, 2.79)	2.36 (1.89, 2.94)			1.93 (1.63, 2.29)	2.05 (1.71, 2.44)	0.29
Upper limbs naevi			2.50 (2.00, 3.14)	2.58 (2.04, 3.27)			3.18 (2.56, 3.96)	3.20 (2.56, 3.98)	0.06
Lower limbs naevi			2.45 (1.93, 3.12)	2.26 (1.77, 2.89)			3.39 (2.64, 4.34)	3.21 (2.50, 4.11)	0.04
**Dysplastic naevi**									
***Categories***									
0	245 (55)	229 (77)	1.00	1.00	689 (72)	458 (92)	1.00	1.00	0.04
1	48 (11)	34 (11)	1.41 (0.86, 2.32)	1.34 (0.80, 2.24)	124 (13)	28 (6)	2.80 (1.82, 4.31)	2.71 (1.75, 4.19)	
≥2	150 (34)	34 (11)	3.90 (2.54, 5.99)	4.06 (2.61, 6.30)	138 (15)	13 (3)	6.44 (3.57, 11.61)	6.03 (3.33, 10.92)	
***Continuous***									
OR per 1 dysplastic naevi			1.20 (1.11, 1.30)	1.20 (1.11, 1.30)			1.78 (1.47, 2.17)	1.74 (1.43, 2.11)	<.0001
**Naevi >5 mm**									
***Categories***									
0	51 (12)	92 (34)	1.00	1.00	405 (46)	324 (68)	1.00	1.00	0.50
1–2	65 (15)	72 (27)	1.68 (1.02, 2.75)	1.72 (1.04, 2.85)	313 (35)	126 (26)	1.91 (1.48, 2.46)	1.83 (1.41, 2.37)	
>2	306 (73)	104 (39)	5.46 (3.55, 8.40)	5.02 (3.23, 7.79)	164 (19)	30 (6)	4.25 (2.80, 6.46)	3.91 (2.56, 5.98)	
***Continuous***									
Whole‐body OR per 1 naevi >5 mm††	9 (2, 22)	2 (0, 5)	1.06 (1.04, 1.08)	1.06 (1.04, 1.07)	1 (0, 2)	0 (0, 1)	1.28 (1.20, 1.38)	1.26 (1.18, 1.36)	<.0001
Whole‐body OR per adjusted SD increase in naevi >5 mm‡‡			2.01 (1.55, 2.60)	1.87 (1.43, 2.43)			1.86 (1.56, 2.23)	1.79 (1.49, 2.14)	0.93

CI, confidence interval; IQR, interquartile range; OR, odds ratio; SD, standard deviation.

Data were missing for participants who did not have a clinical skin examination (353 in Australia, 876 in Leeds). In addition, data were missing for Leeds for trunk (1), upper limbs (1) and lower limbs (7).

†Models adjusted for age (continuous), sex and city of recruitment in Australia.

‡Further adjusted for pigmentation score and hair colour.

§*P*‐value for the interaction between naevus phenotype and population (Australia/Leeds) using minimally adjusted models.

¶*P*‐value based on model excluding the top category

††OR per 1‐unit increase in naevus count modelled as a continuous variable.

‡‡OR per adjusted standard deviation, stratified by country (Australia/Leeds) and adjusted for age (5‐year groups) and sex, using the OPERA method.^22^

Risk of melanoma increased sharply with increasing number of clinically assessed naevi (Table 4). The top category of >200 naevi could only be assessed in the Australian sample as 50% and 16% of Australian cases and controls, respectively, were in this category, compared with 4% and 0% of Leeds cases and controls (5% and 0% of Leeds cases and controls ≤40 years; Table S2). For both countries, fewer naevi occurred on the head and neck than on other body sites, but the OR for melanoma per additional naevus was higher for head and neck naevi. Based on the ORs per adjusted standard deviation increase in naevi, the upper and lower limbs were the body sites that were most predictive of melanoma risk for Leeds, and the upper and lower limbs and the trunk for Australia. The number of clinically assessed common naevi analysed on a continuous scale, and the presence and number of clinically assessed dysplastic naevi, was each associated with greater relative risks for melanoma in Leeds than in Australia (*P*‐interaction <0.05). This pattern of a higher relative risk of melanoma in Leeds was consistent for common naevi on different body sites when modelled as an OR per 1 naevus increase, and for naevi on the upper and lower limbs when modelled as an OR per adjusted standard deviation.

Since the Australian study recruited only participants aged <40 years, we further examined the naevi results for the Leeds sample in age‐stratified analysis (≤40, >40 years; Table S2). Leeds’ participants aged ≤40 years had higher numbers of naevi than those aged > 40 years; the median total body count was 53 and 23 naevi for younger cases and controls, respectively, and 35 and 15 naevi for older cases and controls, respectively. This increase was more noticeable on the trunk, with 17 and 10 naevi for younger cases and controls, respectively, and 9 and 5 naevi for older cases and controls, respectively. Nevertheless, similar ORs between naevus counts and melanoma risk were observed for the Leeds study when stratified by age (≤40 years, >40 years) and there was no evidence of interaction by age (all *P*‐interaction values were ≥0.15; Table S2). In analyses stratified by sex (Table S3), we found that higher naevus counts on the head and neck were associated with a stronger relative risk for melanoma for women than men, and this was consistent across countries: in Australia the OR per adjusted SD increase in naevi was 2.19 (95% CI 1.63, 2.95) for women and 1.59 (95% CI 1.20, 2.11) for men (*P*‐interaction = 0.03), and in Leeds was 1.92 (1.57, 2.35) for women and 1.29 (1.06, 1.57) for men (*P*‐interaction =0.01).

Table S4 shows the association of naevi with melanoma risk, stratified by pigmentation score and hair colour. In Leeds, there was evidence of a stronger association between clinically assessed naevi and melanoma risk for participants with a sun‐sensitive phenotype, but the opposite was observed in Australia, whereby self‐reported naevi were a stronger risk factor for those with a sun‐resistant phenotype. Stratified by hair colour, the association of melanoma risk with clinically assessed naevi (Table S4) and sun‐sensitive pigmentation phenotype (Table S5) appeared stronger for participants with red hair, although the confidence intervals were wide. There was no evidence for interactions of dysplastic naevi with common naevi or hair colour on melanoma risk (data not shown).

## Discussion

The findings from these two population‐based case–control studies, using the same measurement protocols and harmonized data, allow a direct comparison of the magnitude of associations of pigmentary and naevus phenotype with melanoma risk in two countries with similar ethnic background but vastly different ambient sun exposure.

The emergence of naevi is thought to be under strong genetic control, whereas sun exposure influences the mean number of naevi.^7^ As naevus measurement and training protocols were essentially the same across our Leeds and Australian studies and the samples had a similar genetic background,^23^ we can reasonably assume that the observed large (approximately threefold, age‐adjusted) differences in clinically assessed number of naevi are due to higher sun exposure in Australia than the UK. Similarly, the proportion of participants with one or more dysplastic naevi or with large naevi was also higher in Australia than Leeds. Bataille and colleagues’ smaller, clinic‐based, cross‐country comparison of naevi recruited between 1989–1993 found about twofold greater number of common and dysplastic naevi in Australia than the UK.^9^

A potential limitation of our analysis was the different age structure between studies. There are limited prospective data on naevus counts over time, but it is thought that number of naevi may change with age or cohort effects, and we observed higher numbers of naevi for Leeds’ participants aged ≤40 years than for those aged >40 years. We addressed this in several ways. Firstly, we adjusted all analyses for age. Secondly, we conducted sensitivity analyses stratified by age group (≤40, >40 years); this still showed 3.8 times higher common naevus counts ≥2 mm for Australian cases and 3 times higher for Australian controls, and that the associations of naevi with melanoma risk was similar for younger and older age groups in Leeds. Finally, we also estimated the OR for melanoma per adjusted standard deviation of naevus counts as a way of comparing the predictive strength of this risk factor across the two countries while accounting for the different naevus, age and sex distributions.^22^ Another limitation was the potential bias from the targeted selection of thicker melanomas in the later years of recruitment in the Leeds group. People with thicker melanomas tended to have fewer naevi, but this is also confounded with age, as older people were more likely to have thicker melanomas and fewer naevi.

Interestingly, participants’ perceptions of their own naevus density (using the self‐reported naevus categories), when compared to clinical counts, differed by country and disease status. This indicates that people may report their own naevus phenotype based on how it compares with the ‘norm’ for their peers. Thus, self‐reported naevus density categories should not be used to infer the same absolute naevus counts across different populations.

A meta‐analysis of 49 studies^13^ estimated that the RR for melanoma was 1.02 (95% CI 1.01–1.02) for each additional common naevus, and for people with ≥1 atypical naevi the summary RR was 3.63 (95% CI 2.85–4.62) compared to no atypical naevi. These summary estimates fall in the middle of the estimates for Australia and Leeds; based on absolute counts measured clinically, the relative risk of melanoma ‘per naevus’ was greater in Leeds than in Australia. However, the relative risks for melanoma were similar for the two countries when using the self‐reported naevus categories because the reference group reflected different absolute numbers of naevi in Leeds and Australia. The higher relative risk for melanoma ‘per naevus’ (based on clinical counts) in Leeds indicate that naevi may be a stronger indicator of a genetic predisposition in the UK based on less opportunity for sun exposure to influence naevus development. A previous pooled analysis found that relative risks for melanoma were fairly similar across latitudes and age groups analysed using study‐specific quantiles.^24^

Calculating the population attributable fraction (PAF)^13^ from our study indicates that 64% of cases in Australia and 16% of cases in Leeds were attributable to having >100 naevi. Olsen and colleagues’ meta‐analysis^13^ concluded that patients with ≥25 common naevi and/or ≥1 atypical naevi should be managed as high risk since almost half of melanomas occurred in this group.^13^ In our study, 97% of melanoma cases and 81% of controls from Australia, and 70% of cases and 34% of controls from Leeds met this high‐risk criteria. It may not be practicable or cost‐effective to apply the same high‐risk naevus count criteria to different countries, and it is important to also take into account other risk factors.^25^

The upper and lower limbs were the body sites that were most predictive of melanoma risk for Leeds, and for Australia the most predictive sites were the upper and lower limbs and the trunk, based on the ORs per adjusted standard deviation^22^ increase in naevi. We observed that higher naevus counts on the head and neck were associated with a stronger relative risk for melanoma for women than men, whereas Ribero and colleagues found that men had a higher relative risk for melanoma associated with naevi on the legs, arms and head and neck.^26^

Our relative risk estimates for the associations of pigmentary phenotype factors with melanoma risk for Australia and Leeds were consistent with a previous meta‐analysis.^12^ Based on our findings, the population attributable fraction for red hair colour was 9% in Australia and Leeds, and for very fair skin was 11% and 16%, respectively. The PAFs calculated in the meta‐analysis from weighted averages across the studies were 10% for red hair and 10% for very fair skin.^12^

Some studies have observed super‐multiplicative joint effects of naevi and red hair colour on melanoma risk.^8^, ^27^ There was some suggestion of similar effect modification in our study between naevi and hair colour or pigmentation score, but the findings were not always consistent. Our results suggest that, in most cases, pigmentary and naevus risk factors act independently of each other.

In conclusion, hair and skin colour were the strongest pigmentary phenotype risk factors, and all associations of pigmentary phenotype with melanoma risk were similar across countries. On average, Australians have about three times as many naevi as those living in the UK, which contributes to Australia's higher burden of melanoma. The magnitude of associations for naevus phenotype with melanoma risk was similar for both populations when based on self‐reported measures but differed when based on clinically assessed number of naevi. Personal perceptions of naevus number also differed by country. Self‐reported naevus count density is a consistent and strong risk factor across populations and is suitable for stratifying levels of melanoma risk; however, caution is needed when meta‐analysing data from different countries or when inferring absolute naevus counts from these categories. Classifying people at high risk of melanoma based on their number of naevi should ideally take into account their country of residence, type of counts (clinical or self‐reported), body site on which the naevus counts are measured and sex.

## Supporting information


**Data S1** Creation of a pigmentation score using factor analysis.
**Table S1** A**.** Spearman rank correlations between pigmentary phenotype variables. B. Factor analysis loadings, derived from controls, for creation of a pigmentation score variable including hair colour. C. Subsequent factor analysis *excluding hair colour*. One factor was retained (pigmentation score), which explained 42% of the variance.
**Table S2** Association of clinically‐assessed naevus phenotype with melanoma risk in the Leeds case‐control study, stratified by age ≤ 40, >40 years.
**Table S3** Association of naevus phenotype with melanoma risk in the Australian Melanoma Family Study and Leeds case‐control study, stratified by sex.
**Table S4** Associations of naevus phenotype with melanoma risk in the Australian Melanoma Family Study and Leeds case‐control study, stratified by pigmentation score and hair colour.
**Table S5** Association of pigmentation score with melanoma risk in the Australian Melanoma Family Study and Leeds case‐control study, stratified by hair colour.
