# Supplementary material for: Associations of pigmentary and naevus phenotype with melanoma risk in two populations with comparable ancestry but contrasting levels of ambient sun exposure
Source: J Eur Acad Dermatol Venereol. 2019 Jun 7;33(10):1874–85. doi: 10.1111/jdv.15680 (PMC6800761; doi:10.1111/jdv.15680)
Supplement: Supplementary file 1 — Data S1 Creation of a pigmentation score using factor analysis. Table S1 A. Spearman rank correlations between pigmentary phenotype variables. B. Factor analysis loadings, derived from controls, for creation of a pigmentation score variable including hair colour. C. Subsequent factor analysis excluding hair colour. One factor was retained (pigmentation score), which explained 42% of the variance. Table S2 Association of clinically‐assessed naevus phenotype with melanoma risk in the Leeds case‐control study, stratified by age ≤ 40, >40 years. Table S3 Association of naevus phenotype with melanoma risk in the Australian Melanoma Family Study and Leeds case‐control study, stratified by sex. Table S4 Associations of naevus phenotype with melanoma risk in the Australian Melanoma Family Study and Leeds case‐control study, stratified by pigmentation score and hair colour. Table S5 Association of pigmentation score with melanoma risk in the Australian Melanoma Family Study and Leeds case‐control study, stratified by hair colour. [file JDV-33-1874-s001.docx]

**Supplementary online-only tables and text**

**Creation of a pigmentation score using factor analysis**

We created a pigmentation score using factor analysis to use as an adjustment factor and for stratified analyses. The score summarises the contribution of five correlated, well-established and independent phenotypic variables: skin colour, eye colour, hair colour, childhood freckling, and skin phototype (tanning and burning skin reaction to the sun). The factor analysis was performed using data from controls only, in order to derive the pigmentation score independently of case-control status. We used a polychoric correlation matrix and maximum likelihood with an orthogonal varimax rotation with Kaiser normalisation^1^ to estimate the factors, treating the variables as ordinal. Hair colour was ordered as red, fair or blonde, light brown, and dark brown or black. Eye colour was ordered as blue or grey, green or hazel, and brown or black. We used a parallel analysis technique^2^ to determine the number of factors to retain. Spearman correlations between different phenotype variables were mostly in the range of 0.2-0.3 (see online Supplementary Table S1-A). The initial factor analysis showed that two factors explained 55% of the common variance in the pigmentary variables (31% for factor 1 and 24% for factor 2) (see online Supplementary Table S1-B). Factor 1 loaded heavily on skin colour (0.82) and skin phototype (0.76), whereas factor 2 had a loading of 0.99 for hair colour and correlated perfectly. Since factor 2 was clearly estimating hair colour as the latent trait, we subsequently used reported hair colour in lieu of factor 2. Factor analysis was thus re-run without hair colour and similar loadings were found for the first factor, herein referred to as ‘pigmentation score’ (skin colour: 0.85, skin phototype 0.77, childhood freckling 0.51, eye colour 0.30) (see online Supplementary Table S1-C). The loadings had a similar pattern when performed separately for Australia and Leeds, thus the factor analysis was performed on the combined controls from Australia and Leeds. The proportion of the variance explained by Factor 1 (pigmentation score) was 42%.

**Table S1-A**. Spearman rank correlations between pigmentary phenotype variables

|  | Hair colour | Eye colour | Skin colour | Freckles child | Skin phototype |
| --- | --- | --- | --- | --- | --- |
| Hair colour | 1 |  |  |  |  |
| Eye colour | 0.250 | 1 |  |  |  |
| Skin colour | 0.289 | 0.186 | 1 |  |  |
| Freckles child | 0.234 | 0.062 | 0.235 | 1 |  |
| Skin phototype | 0.251 | 0.119 | 0.410 | 0.279 | 1 |

**Table S1-B**. Factor analysis loadings, derived from controls, for creation of a pigmentation score variable including hair colour

| **Loadings** | **Factor 1** | **Factor 2** |
| --- | --- | --- |
| **Australia and Leeds combined** |  |  |
| Hair colour | 0.153 | 0.988 |
| Eye colour | 0.238 | 0.303 |
| Skin colour | 0.815 | 0.190 |
| Freckles child | 0.478 | 0.223 |
| Skin phototype | 0.761 | 0.154 |

**Table S1-C)** Subsequent factor analysis *excluding hair colour*. One factor was retained (pigmentation score), which explained 42% of the variance.

| **Loadings** | **Factor 1 (pigmentation score)** |
| --- | --- |
| **Australia and Leeds combined** |  |
| Eye colour | 0.304 |
| Skin colour | 0.847 |
| Freckles child | 0.514 |
| Skin phototype | 0.766 |
| **Australia only** |  |
| Eye colour | 0.354 |
| Skin colour | 0.914 |
| Freckles child | 0.588 |
| Skin phototype | 0.826 |
| **Leeds only** |  |
| Eye colour | 0.243 |
| Skin colour | 0.849 |
| Freckles child | 0.403 |
| Skin phototype | 0.689 |
|  |  |

**Table S2**. Association of clinically-assessed **naevus phenotype with melanoma risk** in the Leeds case-control study, **stratified by age ≤ 40, >40 years**

|  | **Leeds age ≤ 40 (N=294)** | | | | **Leeds age >40 (N=1,309)** | | | |  |
| --- | --- | --- | --- | --- | --- | --- | --- | --- | --- |
| **Naevi** | **Case N (%) or median (IQR)** | **Control N (%) or median (IQR)** | **OR (95% CI)^1^** | **Adjusted OR (95% CI)^2^** | **Case N (%) or median (IQR)** | **Control N (%) or median (IQR)** | **OR (95% CI)^1^** | **Adjusted OR (95% CI)^2^** | **P-int^3^** |
| **Naevi on the whole body = 2mm** |  |  |  |  |  |  |  |  |  |
| ***Categories*** |  |  |  |  |  |  |  |  |  |
| 0-15 | 17 (9) | 25 (40) | 1.00 | 1.00 | 176 (23) | 233 (53) | 1.00 | 1.00 | 0.52^4^ |
| 16-40 | 55 (28) | 17 (27) | 4.91 (2.11, 11.42) | 4.26 (1.80, 10.09) | 236 (31) | 146 (33) | 2.22 (1.66, 2.97) | 2.34 (1.73, 3.15) |  |
| 41-60 | 38 (19) | 12 (19) | 4.42 (1.75, 11.17) | 4.03 (1.55, 10.51) | 110 (15) | 28 (6) | 5.50 (3.44, 8.77) | 5.86 (3.64, 9.44) |  |
| 61-80 | 18 (9) | 5 (8) | 4.48 (1.35, 14.85) | 2.95 (0.84, 10.34) | 63 (8) | 16 (4) | 5.55 (3.08, 10.01) | 6.27 (3.43, 11.46) |  |
| 81-100 | 24 (12) | 1 (2) | 36.21 (4.41, 297.55) | 22.35 (2.65, 188.53) | 42 (6) | 4 (1) | 14.87 (5.20, 42.48) | 14.58 (5.07, 41.90) |  |
| 101-200 | 37 (19) | 3 (5) | 17.57 (4.58, 67.35) | 13.28 (3.33, 52.97) | 99 (13) | 9 (2) | 15.73 (7.66, 32.32) | 15.58 (7.53, 32.22) |  |
| >= 201 | 10 (5) | 0 (0) | n/a | n/a | 26 (3) | 0 (0) | n/a | n/a |  |
| ***Quartiles*** |  |  |  |  |  |  |  |  |  |
| Q1 (AMFS: 0-29; Leeds: 0-7) | 6 (3) | 12 (19) | 1.00 | 1.00 | 69 (9) | 122 (28) | 1.00 | 1.00 | 0.55 |
| Q2 (AMFS: 30-69; Leeds: 8-15) | 11 (6) | 13 (21) | 1.60 (0.43, 5.96) | 1.71 (0.45, 6.53) | 107 (14) | 111 (25) | 1.73 (1.16, 2.57) | 1.93 (1.28, 2.92) |  |
| Q3 (AMFS: 70-155; Leeds: 16-29) | 31 (16) | 12 (19) | 4.95 (1.45, 16.83) | 4.42 (1.26, 15.52) | 154 (20) | 109 (25) | 2.59 (1.76, 3.82) | 2.93 (1.95, 4.39) |  |
| Q4 (AMFS: >155; Leeds: >29) | 151 (76) | 26 (41) | 11.20 (3.68, 34.08) | 9.16 (2.90, 28.93) | 422 (56) | 94 (22) | 8.47 (5.76, 12.48) | 9.51 (6.37, 14.20) |  |
| ***Continuous variables*** |  |  |  |  |  |  |  |  |  |
| **Median (IQR) & OR per 1 naevi^5^** |  |  |  |  |  |  |  |  |  |
| Whole-body | 53 (30, 97) | 23 (10, 51) | 1.02 (1.01, 1.03) | 1.02 (1.01, 1.03) | 35 (17, 74) | 15 (7, 28) | 1.03 (1.02, 1.03) | 1.03 (1.02, 1.03) | 0.77 |
| Head and neck | 4 (2, 8) | 1 (1, 5) | 1.17 (1.07, 1.28) | 1.18 (1.08, 1.30) | 2 (1, 5) | 1 (1, 3) | 1.14 (1.10, 1.20) | 1.15 (1.10, 1.20) | 0.51 |
| Trunk | 17 (7, 31) | 10 (3, 20) | 1.05 (1.03, 1.08) | 1.05 (1.02, 1.08) | 9 (3, 22) | 5 (2, 10) | 1.04 (1.03, 1.05) | 1.04 (1.03, 1.06) | 0.38 |
| Upper limbs | 16 (7, 31) | 5 (2, 13) | 1.08 (1.04, 1.11) | 1.06 (1.03, 1.10) | 9 (3, 20) | 3 (1, 8) | 1.09 (1.07, 1.11) | 1.10 (1.08, 1.12) | 0.47 |
| Lower limbs | 13 (5, 30) | 3 (1, 12) | 1.05 (1.02, 1.08) | 1.04 (1.02, 1.07) | 10 (4, 23) | 3 (1, 7) | 1.07 (1.05, 1.09) | 1.07 (1.05, 1.08) | 0.24 |
| **OR per adjusted SD increase in naevi^6^** |  |  |  |  |  |  |  |  |  |
| Whole-body | 1 (1, 2) | 0 (0, 1) | 3.67 (2.09, 6.45) | 3.11 (1.78, 5.42) | 1 (0, 1) | 0 (0, 1) | 2.98 (2.37, 3.75) | 3.03 (2.40, 3.82) | 0.45 |
| Head and neck | 1 (0, 2) | 0 (0, 1) | 2.06 (1.37, 3.11) | 2.17 (1.42, 3.31) | 1 (0, 1) | 0 (0, 1) | 1.53 (1.32, 1.78) | 1.54 (1.33, 1.80) | 0.15 |
| Trunk | 1 (0, 2) | 1 (0, 1) | 2.25 (1.46, 3.46) | 2.15 (1.37, 3.38) | 1 (0, 1) | 0 (0, 1) | 1.88 (1.56, 2.27) | 2.02 (1.66, 2.46) | 0.50 |
| Upper limbs | 1 (0, 2) | 0 (0, 1) | 3.22 (1.90, 5.45) | 2.74 (1.66, 4.52) | 1 (0, 1) | 0 (0, 1) | 3.19 (2.51, 4.05) | 3.27 (2.56, 4.17) | 0.98 |
| Lower limbs | 0 (0, 1) | 0 (0, 0) | 3.61 (1.84, 7.09) | 3.15 (1.60, 6.18) | 0 (0, 1) | 0 (0, 0) | 3.33 (2.55, 4.34) | 3.19 (2.44, 4.18) | 0.70 |
|  |  |  |  |  |  |  |  |  |  |
| **Dysplastic naevi** |  |  |  |  |  |  |  |  |  |
| ***Categories*** |  |  |  |  |  |  |  |  |  |
| 0 | 117 (59) | 49 (78) | 1.00 | 1.00 | 572 (76) | 409 (94) | 1.00 | 1.00 | 0.22 |
| 1 | 37 (19) | 9 (14) | 1.60 (0.71, 3.62) | 1.44 (0.62, 3.37) | 87 (12) | 19 (4) | 3.30 (1.98, 5.52) | 3.23 (1.92, 5.43) |  |
| >= 2 | 45 (23) | 5 (8) | 3.41 (1.25, 9.25) | 2.85 (1.02, 7.94) | 93 (12) | 8 (2) | 8.18 (3.90, 17.16) | 7.70 (3.65, 16.23) |  |
| ***Continuous*** |  |  |  |  |  |  |  |  |  |
| OR per 1 dysplastic naevi | 0 (0, 1) | 0 (0, 0) | 1.43 (1.04, 1.96) | 1.35 (0.98, 1.85) | 0 (0, 0) | 0 (0, 0) | 1.93 (1.52, 2.47) | 1.89 (1.48, 2.41) | 0.21 |
|  |  |  |  |  |  |  |  |  |  |
| **Naevi >5mm** |  |  |  |  |  |  |  |  |  |
| ***Categories*** |  |  |  |  |  |  |  |  |  |
| 0 | 79 (42) | 38 (61) | 1.00 | 1.00 | 326 (47) | 286 (68) | 1.00 | 1.00 | 0.67 |
| 1-2 | 73 (38) | 21 (34) | 1.62 (0.86, 3.05) | 1.43 (0.74, 2.75) | 240 (35) | 105 (25) | 1.98 (1.49, 2.62) | 1.91 (1.44, 2.54) |  |
| >2 | 38 (20) | 3 (5) | 6.75 (1.93, 23.66) | 4.91 (1.35, 17.88) | 126 (18) | 27 (6) | 4.04 (2.59, 6.32) | 3.79 (2.42, 5.96) |  |
| ***Continuous*** |  |  |  |  |  |  |  |  |  |
| Whole-body OR per 1 naevi >5mm^5^ | 1 (0, 2) | 0 (0, 1) | 1.39 (1.13, 1.72) | 1.32 (1.07, 1.62) | 1 (0, 2) | 0 (0, 1) | 1.27 (1.18, 1.37) | 1.26 (1.17, 1.35) | 0.53 |
| Whole-body OR per adjusted SD increase in naevi >5mm^6^ | 0 (0, 1) | 0 (0, 0) | 2.60 (1.45, 4.66) | 2.25 (1.26, 4.00) | 0 (0, 1) | 0 (0, 0) | 1.80 (1.49, 2.17) | 1.74 (1.44, 2.10) | 0.35 |
| **Self-reported naevi** |  |  |  |  |  |  |  |  |  |
| None | 6 (2) | 4 (6) | 1.00 | 1.00 | 170 (10) | 93 (21) | 1.00 | 1.00 | 0.62 |
| Few | 86 (26) | 25 (40) | 2.10 (0.54, 8.20) | 1.82 (0.44, 7.65) | 685 (42) | 227 (52) | 1.66 (1.24, 2.23) | 1.76 (1.30, 2.38) |  |
| Some | 156 (48) | 28 (44) | 3.48 (0.91, 13.34) | 3.07 (0.75, 12.62) | 562 (34) | 90 (21) | 3.47 (2.46, 4.91) | 3.74 (2.63, 5.33) |  |
| Many | 78 (24) | 6 (10) | 7.82 (1.69, 36.12) | 6.87 (1.39, 33.90) | 218 (13) | 28 (6) | 4.30 (2.67, 6.93) | 4.41 (2.72, 7.15) |  |

OR, odds ratio; CI, confidence interval; IQR = inter-quartile range; SD, standard deviation.

Data were missing for participants who did not have a clinical skin exam (97 in age<=40, 779 in age>40). In addition, data were missing for total (32, 125), head & neck (32, 125), trunk (32, 126), upper limbs (32, 126), lower limbs (32, 131), dysplastic naevi (32, 125) and naevi>5mm (32, 125).

^1^ Models adjusted for age (continuous) and sex.

^2^ Further adjusted for Factor 1 (pigmentation score) and hair colour.

^3^ P-value for the interaction between naevus phenotype and age-group (≤ 40, > 40 years) based on minimally-adjusted models.

^4^ P-value based on model excluding the top category.

^5^ OR per 1-unit increase in naevus count modelled as a continuous variable.

^6^ OR per adjusted standard deviation, adjusted for age (5-yr groups) and sex, using the OPERA method.^3^

**Table S3**. Association of **naevus phenotype with melanoma risk** in the Australian Melanoma Family Study and Leeds case-control study, **stratified by sex**

|  | **Male** | | | | | **Female** | | | |  |
| --- | --- | --- | --- | --- | --- | --- | --- | --- | --- | --- |
|  | **Australia** | | | | | | | | |  |
| **Naevi** | **Case N (%) or median (IQR)** | **Control N (%) or median (IQR)** | **OR (95% CI)^1^** | **Adjusted OR**  **(95% CI)^2^** | **Case N (%) or median (IQR)** | | **Control N (%) or median (IQR)** | **OR (95% CI)^1^** | **Adjusted OR**  **(95% CI)^2^** | **P-int^3^** |
| **Self-reported naevi** |  |  |  |  |  | |  |  |  |  |
| None | 11 (5) | 19 (9) | 1.00 | 1.00 | 10 (3) | | 21 (8) | 1.00 | 1.00 | 0.55 |
| Few | 71 (31) | 105 (49) | 1.22 (0.53, 2.83) | 1.40 (0.59, 3.35) | 101 (27) | | 132 (48) | 1.57 (0.69, 3.56) | 1.67 (0.71, 3.92) |  |
| Some | 97 (42) | 64 (30) | 2.73 (1.17, 6.36) | 3.22 (1.34, 7.74) | 167 (45) | | 94 (34) | 3.82 (1.69, 8.65) | 4.16 (1.77, 9.78) |  |
| Many | 50 (22) | 25 (12) | 3.72 (1.48, 9.39) | 4.27 (1.63, 11.15) | 97 (26) | | 26 (10) | 7.71 (3.16, 18.80) | 8.29 (3.28, 20.92) |  |
| **Median (IQR) & OR per adjusted SD increase in naevi^4^** | | |  |  |  | |  |  |  |  |
| Whole-body naevi | 202 (105, 309) | 75 (40, 157) | 2.76 (1.95, 3.89) | 2.84 (1.97, 4.10) | 200 (108, 305) | | 67 (25, 154) | 2.66 (1.94, 3.66) | 2.62 (1.90, 3.63) | 0.33 |
| Head and neck naevi | 12 (5, 21) | 6 (2, 13) | 1.59 (1.20, 2.11) | 1.72 (1.26, 2.34) | 16 (7, 26) | | 6 (1, 13) | 2.19 (1.63, 2.95) | 2.39 (1.74, 3.29) | **0.03** |
| Trunk naevi | 58 (31, 90) | 24 (9, 52) | 2.30 (1.68, 3.15) | 2.51 (1.78, 3.54) | 38 (18, 63) | | 15 (5, 34) | 2.34 (1.74, 3.16) | 2.41 (1.77, 3.27) | 0.35 |
| Upper limbs naevi | 71 (31, 112) | 27 (13, 57) | 2.62 (1.86, 3.70) | 2.77 (1.92, 4.00) | 79 (38, 124) | | 26 (10, 57) | 2.58 (1.89, 3.53) | 2.66 (1.92, 3.68) | 0.27 |
| Lower limbs naevi | 49 (23, 80) | 16 (5, 32) | 2.88 (1.97, 4.22) | 2.59 (1.76, 3.81) | 54 (27, 99) | | 13 (5, 49) | 2.20 (1.61, 3.01) | 2.08 (1.51, 2.86) | 0.80 |
|  |  |  |  |  |  | |  |  |  |  |
|  | **Leeds** | | | | | | | | | |
| **Self-reported naevi** |  |  |  |  |  | |  |  |  |  |
| None | 72 (9) | 42 (21) | 1.00 | 1.00 | 104 (9) | | 55 (18) | 1.00 | 1.00 | 0.84 |
| Few | 323 (38) | 99 (49) | 1.88 (1.21, 2.93) | 2.01 (1.27, 3.16) | 448 (40) | | 153 (51) | 1.55 (1.06, 2.26) | 1.59 (1.07, 2.35) |  |
| Some | 310 (37) | 46 (23) | 3.75 (2.26, 6.22) | 4.16 (2.48, 6.98) | 408 (36) | | 72 (24) | 2.99 (1.95, 4.60) | 3.21 (2.06, 5.00) |  |
| Many | 137 (16) | 16 (8) | 4.77 (2.48, 9.17) | 4.98 (2.57, 9.65) | 159 (14) | | 18 (6) | 4.66 (2.54, 8.55) | 4.84 (2.60, 8.99) |  |
| **Median (IQR) & OR per adjusted SD increase in naevi^4^** | | |  |  |  | |  |  |  | \|  \| \| --- \| |
| Whole-body naevi | 41 (20, 84) | 16 (7, 29) | 2.59 (1.92, 3.49) | 2.59 (1.92, 3.51) | 39 (18, 77) | | 14 (7, 29) | 3.59 (2.66, 4.83) | 3.54 (2.62, 4.79) | 0.15 |
| Head and neck naevi | 2 (1, 4) | 1 (0, 3) | 1.29 (1.06, 1.57) | 1.28 (1.05, 1.56) | 3 (1, 7) | | 2 (1, 3) | 1.92 (1.57, 2.35) | 2.02 (1.64, 2.49) | **0.01** |
| Trunk naevi | 18 (7, 36) | 7 (2, 14) | 2.14 (1.62, 2.84) | 2.20 (1.65, 2.94) | 8 (3, 17) | | 4 (1, 9) | 1.80 (1.45, 2.24) | 1.94 (1.55, 2.43) | 0.30 |
| Upper limbs naevi | 9 (4, 18) | 3 (1, 7) | 2.65 (1.94, 3.60) | 2.66 (1.95, 3.63) | 11 (4, 24) | | 3 (1, 9) | 3.70 (2.74, 5.01) | 3.71 (2.72, 5.04) | 0.15 |
| Lower limbs naevi | 9 (3, 21) | 2 (1, 6) | 3.18 (2.18, 4.63) | 3.06 (2.10, 4.46) | 11 (4, 29) | | 3 (1, 7) | 3.63 (2.60, 5.07) | 3.41 (2.44, 4.76) | 0.60 |

OR, odds ratio; CI, confidence interval; IQR = inter-quartile range; SD, standard deviation.

^1^ Models adjusted for age (continuous), sex, and city of recruitment in Australia.

^2^ Further adjusted for pigmentation score and hair colour.

^3^ P-value for the interaction between naevus phenotype and sex based on minimally-adjusted models.

^4^ OR per adjusted standard deviation, stratified by country (Australia/Leeds) and adjusted for age (5-yr groups) and sex, using the OPERA method.^3^

**Table S4:** Associations of **naevus phenotype with melanoma risk** in the Australian Melanoma Family Study and Leeds case-control study**, stratified by pigmentation score and hair colour**

|  | **Clinically-assessed whole-body naevi ≥ 2mm** | |  | **Self-reported naevus density** | | | |
| --- | --- | --- | --- | --- | --- | --- | --- |
|  | OR (95% CI) per adjusted SD increase in naevi^2^ | P-interaction^3^ |  | OR (95% CI) | | | |
|  |  |  |  | None/Few^4^ | Some | Many | P-interaction^3^ |
| **Pigmentation Score**^1^ | |  |  |  |  |  |  |
| **Australia** |  |  |  |  |  |  |  |
| Tertile 1 | 3.46 (2.09, 5.72) | 0.85 |  | 1.00 | 4.92 (2.70, 8.96) | 5.62 (2.50, 12.64) | 0.03 |
| Tertile 2 | 2.92 (1.87, 4.55) |  |  | 1.00 | 2.62 (1.58, 4.35) | 3.65 (1.88, 7.06) |  |
| Tertile 3 | 2.26 (1.61, 3.17) |  |  | 1.00 | 1.56 (1.02, 2.38) | 3.70 (2.08, 6.61) |  |
|  |  |  |  |  |  |  |  |
| **Leeds** |  |  |  |  |  |  |  |
| Tertile 1 | 2.26 (1.65, 3.09) | 0.02 |  | 1.00 | 2.46 (1.58, 3.84) | 4.21 (1.98, 8.96) | 0.34 |
| Tertile 2 | 3.07 (2.14, 4.39) |  |  | 1.00 | 1.59 (1.08, 2.35) | 2.22 (1.19, 4.15) |  |
| Tertile 3 | 5.77 (3.48, 9.58) |  |  | 1.00 | 2.87 (1.86, 4.41) | 3.35 (1.73, 6.48) |  |
|  |  |  |  |  |  |  |  |
| **Hair colour** |  |  |  |  |  |  |  |
| **Australia** |  |  |  |  |  |  |  |
| Dark brown/black | 3.20 (2.11, 4.86) | 0.82 |  | 1.00 | 2.23 (1.38, 3.61) | 3.30 (1.68, 6.48) | 0.09 |
| Light brown | 2.39 (1.72, 3.32) |  |  | 1.00 | 3.61 (2.30, 5.66) | 5.63 (3.19, 9.97) |  |
| Fair or blonde | 2.49 (1.37, 4.53) |  |  | 1.00 | 3.07 (1.47, 6.42) | 5.94 (2.26, 15.64) |  |
| Red | 6.45 (1.15, 36.02) |  |  | 1.00 | 0.50 (0.18, 1.43) | 2.76 (0.29, 26.67) |  |
| **Leeds** |  |  |  |  |  |  |  |
| Dark brown/black | 2.84 (2.14, 3.77) | 0.44 |  | 1.00 | 2.60 (1.87, 3.62) | 2.50 (1.53, 4.08) | 0.53 |
| Light brown | 3.20 (2.07, 4.93) |  |  | 1.00 | 1.93 (1.20, 3.09) | 5.51 (2.28, 13.34) |  |
| Fair or blonde | 2.50 (1.49, 4.20) |  |  | 1.00 | 2.14 (1.15, 4.00) | 3.28 (1.10, 9.77) |  |
| Red | 7.51 (2.35, 24.03) |  |  | 1.00 | 1.97 (0.76, 5.14) | 4.47 (0.57, 34.87) |  |

OR, odds ratio; CI, confidence interval; SD, standard deviation.

^1^ Based on factor analysis of polychoric correlations of four ordinal pigmentary phenotype variables. Tertile cut-points were based on the combined control distribution.

^2^ OR per adjusted standard deviation, stratified by country (Australia/Leeds) and adjusted for age (5-yr groups) and sex, using the OPERA method.^22^

^3^ From an interaction term between pigmentation score/hair colour and naevi in the model.

**Table S5:** Association of pigmentation score with melanoma risk in the Australian Melanoma Family Study and Leeds case-control study, stratified by hair colour

| **Hair colour** | **Pigmentation Score^1^** | | | |
| --- | --- | --- | --- | --- |
|  | OR (95% CI)^2^ | | | |
|  | Tertile 1 | Tertile 2 | Tertile 3 | P-interaction^3^ |
| **Australia** |  |  |  |  |
| Dark brown/black | 1.00 | 1.42 (0.83, 2.45) | 1.50 (0.88, 2.54) | 0.05 |
| Light brown | 1.00 | 1.41 (0.83, 2.39) | 0.88 (0.53, 1.47) |  |
| Fair or blonde | 1.00 | 2.45 (0.91, 6.62) | 3.84 (1.50, 9.84) |  |
| Red | 1.00 | n/a | 12.39 (2.99, 51.43) |  |
| **Leeds** |  |  |  |  |
| Dark brown/black | 1.00 | 1.11 (0.81, 1.52) | 1.85 (1.29, 2.67) | 0.55 |
| Light brown | 1.00 | 1.78 (1.08, 2.94) | 2.00 (1.20, 3.34) |  |
| Fair or blonde | 1.00 | 1.91 (0.93, 3.90) | 2.09 (1.07, 4.08) |  |
| Red | 1.00 | 1.37 (0.23, 8.22) | 2.24 (0.42, 11.90) |  |

OR, odds ratio; CI, confidence interval

^1^ Based on factor analysis of polychoric correlations of four ordinal pigmentary phenotype variables. Tertile cut-points were based on the combined control distribution.

^2^ Model adjusted for age, sex, and city of recruitment in Australia, and stratified by hair colour.

^3^ From an interaction term between pigmentation score and hair colour in the model. In Australia it was generated without the red hair group.

**References**

1. Kaiser HF. The varimax criterion for analytic rotation in factor analysis. *Psychometrika* 1958;**23**:187-200.

2. Ledesma RD, Valero-Mora P. Determining the Number of Factors to Retain in EFA: an easy-to-use computer program for carrying out Parallel Analysis. *Practical Assessment, Research & Evaluation* 2007;**12**.

3. Hopper JL. Odds per adjusted standard deviation: comparing strengths of associations for risk factors measured on different scales and across diseases and populations. *Am J Epidemiol* 2015;**182**:863-7.
